# The Effect of Cefazolin on the Gut Microbiome of Female Rats After Spinal Cord Injury

**DOI:** 10.3390/microorganisms13102324

**Published:** 2025-10-07

**Authors:** Luis H. Pagán-Rivera, Filipa Godoy-Vitorino, Natalie M. Meléndez-Vázquez, Samuel E. Ocasio-Rivera, María E. Santiago-Gascot, Jose M. Santiago, Iris Salgado, Viviana González, Osmarie Martínez-Guzmán, Mauricio Cáceres-Chacón, Aranza Torrado-Tapias, Jorge D. Miranda

**Affiliations:** 1 Department of Physiology, University of Puerto Rico, Medical Sciences Campus, San Juan, PR 00936, USA; 2 Department of Microbiology and Medical Zoology, University of Puerto Rico, Medical Sciences Campus, San Juan, PR 00936, USA; 3 Department of Natural Sciences, University of Puerto Rico, Carolina Campus, Carolina, PR 00984, USA; 4 School of Medicine, Universidad Central del Caribe, Bayamon, PR 00956, USA; 5 Department of Anatomy and Neurobiology, University of Puerto Rico, Medical Sciences Campus, San Juan, PR 00936, USA; 6 National Human Genome Research Institute, National Institutes of Health, Bethesda, MD 20892, USA

**Keywords:** trauma, microbiota, antibiotics, dysbiosis

## Abstract

Spinal cord injury (SCI) is a devastating neurological state that could lead to motor, sensory, and autonomic dysfunction. In addition to its direct impact on the central nervous system, SCI exerts systemic effects, including disruption of gut homeostasis and alterations in the gut microbiota, which can contribute to sustained inflammation and hinder functional recovery. While antibiotic administration during the acute phase of SCI is clinically indicated, it may exacerbate microbial dysbiosis. In this study, we investigate the combined effects of SCI and cefazolin treatment on the gut microbiome of female rats. Animals were assigned to three groups: NAÏVE (no intervention), SHAM (cefazolin only), and INJURY (T10 spinal cord contusion plus cefazolin). Cefazolin was administered for seven days after the injury, fecal samples were collected at baseline (day 0), and on days 7, 14, 21, and 28 post-SCI. DNA was extracted and subjected to 16S rRNA gene amplicon sequencing, followed by bioinformatic analysis. Our findings revealed significant microbial dysbiosis in the INJURY group, including reduced alpha diversity and distinct shifts in microbial composition. These changes were most prominent during the acute phase post-SCI. Our findings highlight the compounding effects of spinal trauma and antibiotic exposure on the gut microbiome and suggest that maintaining microbial stability may represent a promising avenue to support recovery after SCI.

## 1. Introduction

Damage to the spinal cord results in a detrimental neurological state [1]. The disruption of neuronal axons impairs communication in the central nervous system (CNS) and the autonomic nervous system (ANS), resulting in paralysis and long-term dysfunction across multiple physiological systems [2]. Paralysis and sensory impairment are severe outcomes of spinal cord injury (SCI), which lead to lifelong nervous system disease, mainly due to the scarcity of viable treatments [3]. The average lifetime cost of care for a SCI patient exceeds USD 1 million and can surpass USD 3 million, depending on the individual’s age and the severity of the injury [4]. The high incidence rate, associated disability, and substantial costs of care can place a significant economic and medical burden on both the patient’s family and society, making SCI an increasingly pressing concern for public health [5]. Furthermore, individuals affected also face significant challenges such as bladder and bowel dysfunction, heightened susceptibility to infections, depression, anxiety, fatigue, impaired temperature regulation, and an increased risk of cardiovascular complications and disease [6]. Experimental and clinical evidence indicate that SCI can severely impair multiple organ systems, including the gastrointestinal (GI) tract, lungs, kidneys, heart, and liver, impairing organ function and diminishing quality of life (QoL) for individuals with SCI [7,8,9,10]. In recent years, research has revealed that SCI induces systemic immune disturbances and inflammatory responses, profoundly affecting the body’s adaptive processes and metabolic balance [11,12].

Gastrointestinal (GI) complications, including altered motility, constipation, and inflammation, are prevalent in up to 60% of SCI patients and are considered a serious problem compromising QoL [13]. Although SCI has a significant impact on GI function, research on its acute and chronic effects, as well as the underlying mechanisms driving these changes, remains limited. The acute phase of SCI is typically defined as the first moments after the lesion until the first seven days post-injury in rodents, and up to 14 days in humans, a period marked by intense inflammation, neuronal damage, and disruption of systemic homeostasis, including gastrointestinal function [14]. In this context, Herrera et al. studied the vascular changes in the GI system following SCI in both acute and chronic phases using adult male mice. They showed that vascular integrity in the GI tract is compromised early after injury and continues to be affected in the chronic phase [15]. This disruption may affect not only the intestinal epithelium and its integrity, but also the microbes residing in the GI tract.

The gut microbiome is a collection of trillions of microbial species—including bacteria, fungi, and viruses—and is considered the most diverse niche of the human body [16,17]. This complex microbial community provides numerous advantages to its host, including immunologic support, preservation of intestinal barrier integrity, and metabolism of vital nutrients such as vitamins and carbohydrates [18,19,20]. A disruption in the natural state of the microbiome—resulting in the loss of beneficial functions to the host—is termed dysbiosis. This gut imbalance can result from host-specific factors, including genetic predisposition, health conditions (such as infections and inflammation), and lifestyle choices. Additionally, environmental influences, such as diet and continued administration of antibiotics and medications, play a significant role in its development [21]. The rapid advancement of high-throughput sequencing technology has significantly improved the accuracy and efficiency of metagenomic analysis, establishing it as a reliable tool for detecting dysbiosis in the gut microbiome [22]. Key indicators of dysbiosis have been identified, including a decrease in the *Bacillota phylum* (former Firmicutes) with an increase in the Bacteroidetes phylum, altering what is known as the Firmicutes/Bacteroidetes (F/B) ratio [23], and a reduction in anti-inflammatory genera such as *Lactobacillus* and *Bifidobacterium* [24].

Multiple studies have reported the presence of dysbiosis after SCI in both human and rat models [25,26,27,28]. Spinal cord injury can impact sympathetic preganglionic neurons in the thoracic region, disrupting the regulation and stability of postganglionic neurons that innervate the gastrointestinal tract. The loss of neural input to the GI system impairs motility, mucus secretion, immune function, and epithelial barrier integrity, ultimately facilitating bacterial translocation and contributing to gut dysbiosis [29]. The administration of antibiotics helps minimize possible infection after any type of lesion to the spinal cord, and its use is warranted. However, in a 2016 study using mice, Kigerl et al. demonstrated that antibiotic-induced gut dysbiosis may establish a feedback loop that impairs neurological recovery and advances the onset of secondary health complications following SCI [30]. Repeated antibiotic use, physical inactivity, and depression—common in individuals with chronic SCI—can further alter the gut microbiome, intensifying the harmful effects of dysbiosis on metabolism, overall health, and QoL [31,32,33,34]. Understanding the impact of SCI and antibiotic administration as sources of dysbiosis following SCI is essential for developing effective treatments for patients. Moreover, recent advancements in understanding the bidirectional interaction between the gut microbiota and the CNS, known as the gut–brain axis, have introduced new approaches for SCI research, potentially leading to novel treatment developments [35]. Therapeutic approaches targeting the gut–brain axis must account for these dysbiotic alterations to optimize recovery and outcome. Research on the gut microbiome in individuals with SCI remains limited [36], and targeting the microbiota presents a promising therapeutic strategy for enhancing functional recovery and mitigating complications associated with SCI. Here, we provide a detailed microbiome profile analysis of the impact of post-SCI antibiotic administration across multiple timepoints through acute and chronic stages.

## 2. Results

### 2.1. Study Design and Sample Collection

**Figure 1:**
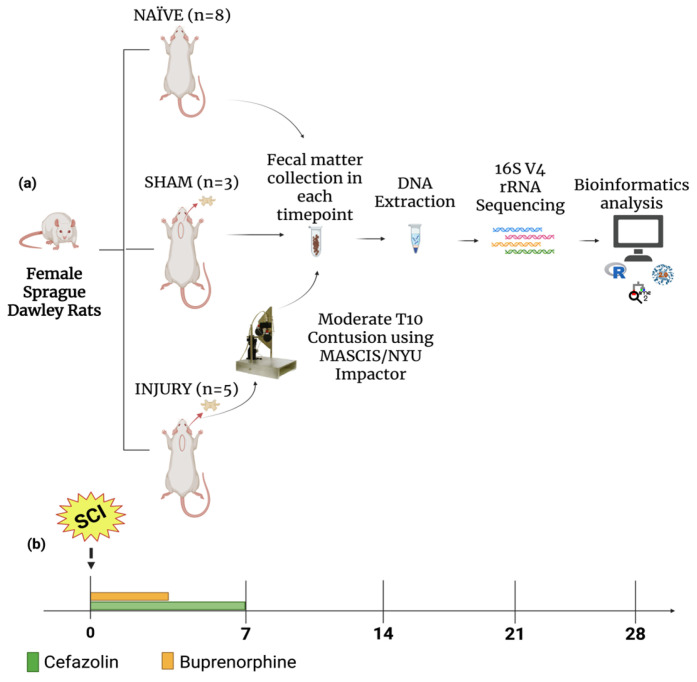
Experimental design. (**a**) Female Sprague Dawley rats were divided into three groups: NAÏVE (*n* = 8), SHAM (*n* = 3), and INJURY (*n* = 5). The NAÏVE animals did not receive any type of intervention. The SHAM and INJURY groups received cefazolin as post-op treatment, with the INJURY group also receiving moderate spinal cord contusion using the MACSIS/NYU Impactor. Fecal samples were collected at multiple timepoints, followed genomic DNA extraction, and 16S rRNA gene sequencing of the V4 region. Downstream microbial analysis was followed using standard bioinformatics pipelines. (**b**) Timeline of sample collection. Cefazolin (green) and buprenorphine (orange) administration was conducted during the acute post-injury period, with fecal samples collected at five timepoints: Days 0, 7, 14, 21, and 28. Created in BioRender. Pagan, L. (2025) https://BioRender.com/4keycgs.

To evaluate the impact of cefazolin administration following spinal cord injury (SCI) on gut microbiome dynamics, we performed a 16S rRNA gene sequencing analysis in fecal samples collected from female Sprague Dawley rats across five timepoints. Animals were randomly assigned to three experimental groups: NAÏVE (*n* = 8), which did not receive any type of intervention, SHAM (*n* = 3), which received only cefazolin, and INJURY (*n* = 5), which received a moderate contusion after a T10 laminectomy followed by the administration of cefazolin. Cefazolin was administered twice daily for seven days in both SHAM and INJURY groups, and fecal pellets were collected on days 0 (before intervention), 7, 14, 21, and 28 post-injury, for a total of 62 fecal samples analyzed (Figure 1a,b).

### 2.2. Beta Diversity Reveals Distinct Clustering Between INJURY and SHAM Groups

**Figure 2:**
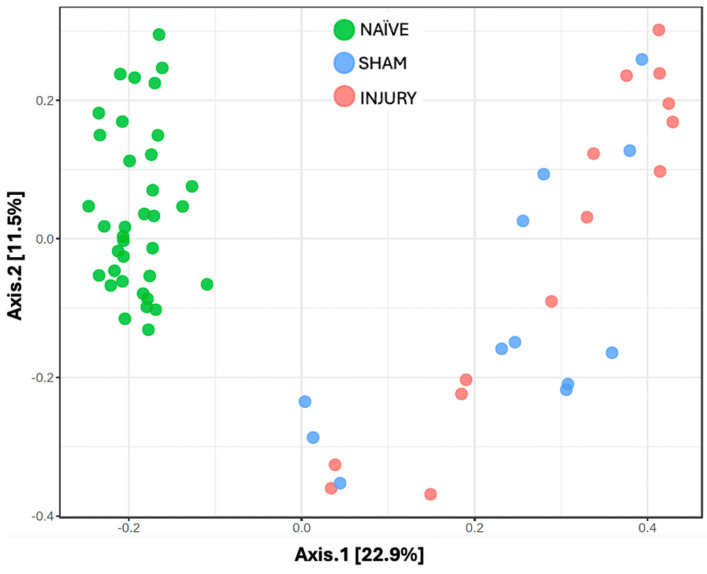
Principal Coordinates Analysis (PCoA) of gut microbiome beta diversity across experimental groups. Beta diversity was assessed using Bray–Curtis dissimilarity and visualized through PCoA to examine differences in microbial community among NAÏVE (green), SHAM (blue), and INJURY (red) groups. Each point represents the microbiota profile of an individual fecal sample. The NAÏVE group clustered distinctly from both SHAM and INJURY groups (PERMANOVA NAÏVE vs. SHAM *p*-value = 0.0015, PERMANOVA NAÏVE vs. INJURY *p*-value = 0.0015), while SHAM and INJURY showed a significant difference in microbial community (PERMANOVA SHAM vs. INJURY *p*-value = 0.045). This indicates that cefazolin administration alone induces microbial community alteration, along with SCI. The overall statistical analysis using PERMANOVA revealed a significant difference in community composition between groups (F = 9.459, R^2^ = 0.243, *p* = 0.001, NAÏVE *n* = 28, SHAM *n* = 15, INJURY *n* = 19).

Beta diversity analyses were conducted to capture inter-sample variability and insight into the distinct microbial communities across treatment conditions to assess differences in overall microbial community composition between experimental groups. This initial analysis included all available samples from each group, regardless of timepoint, thereby providing a broader overview of group-level bacterial differences. Principal Coordinates Analysis (PCoA) based on the Bray–Curtis Dissimilarity Index showcased distinct clustering of bacterial communities between the three experimental groups (Figure 2). The NAÏVE group revealed separate clustering from the SHAM and INJURY groups, while partial overlap was observed between SHAM and INJURY samples. These findings suggest that cefazolin independently alters gut microbial composition, with additional disruptions observed when combined with SCI. Notably, Axis 1 (22.9%) and Axis 2 (11.5%) together explained 34.4% of the total variation in microbial community structure, highlighting meaningful divergence in beta diversity across treatment groups.

### 2.3. Alpha Diversity Is Reduced Following Cefazolin Administration and Spinal Cord Injury

**Figure 3:**
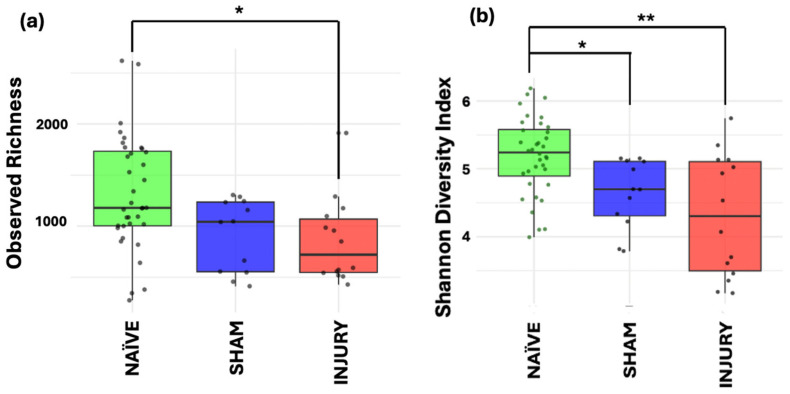
Alpha diversity metrics of gut microbiome across NAÏVE, SHAM, and INJURY groups. Observed Richness (**a**) displayed significantly lower levels in the INJURY group compared with NAÏVE group (Kruskal–Wallis *p* < 0.017), while Shannon diversity (**b**) was significantly reduced in both SHAM (Kruskal–Wallis *p* < 0.012) and INJURY groups (Kruskal–Wallis *p* < 0.002) compared to NAÏVE animals. No significant differences were observed between SHAM and INJURY groups (Kruskal–Wallis *p* > 0.05). Findings indicate that cefazolin administration combined with SCI leads to a reduction in gut microbial diversity. * *p* < 0.05, ** *p* < 0.01. NAÏVE *n* = 28, SHAM *n* = 15, INJURY *n* = 19.

To evaluate within-sample microbial diversity, we performed alpha diversity metrics using two indices. Observed Richness, which measures the number of unique taxa present, and Shannon diversity, which accounts for both richness and relative distribution of the bacterial communities. The INJURY group showed a significant decrease in Observed Richness compared to the NAÏVE group (Kruskal–Wallis *p* < 0.017), indicating a reduction in the number of microbial taxa following cefazolin administration and SCI (Figure 3a). While SHAM animals showed intermediate level richness, no statistically significant difference was observed between SHAM and the other groups (Kruskal–Wallis *p* > 0.05).

Both SHAM (Kruskal–Wallis *p* < 0.012) and INJURY (Kruskal–Wallis *p* < 0.002) groups exhibited a decreased Shannon index, representing decreased diversity (Figure 3b). These findings suggest that the combination of cefazolin administration and/or SCI induces notable reductions in both microbial richness and diversity, revealed by the most apparent effect in the INJURY group. Together, these results point to an altered and less diverse gut microbiome in response to the combination of both cefazolin administration and SCI.

### 2.4. Firmicutes/Bacteroidetes Ratio Is Reduced by Cefazolin and SCI

**Figure 4:**
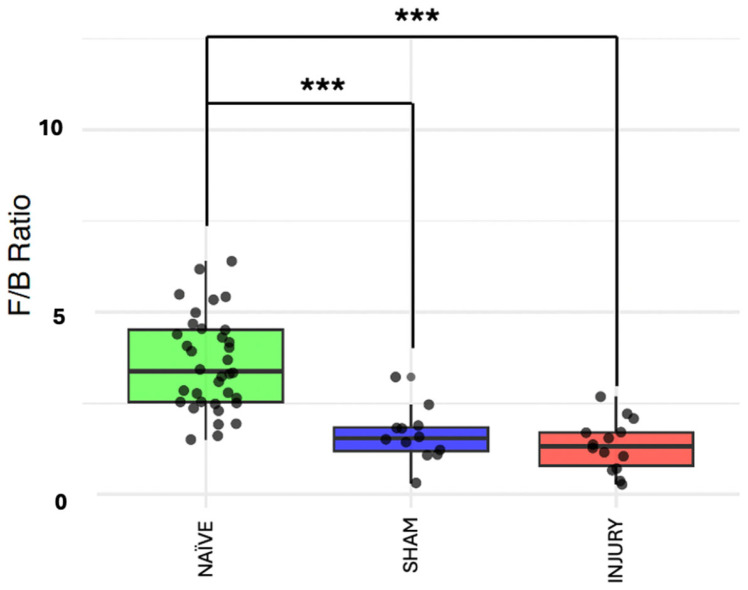
F/B ratio across experimental groups. Boxplots showing the F/B ratio in NAÏVE, SHAM, and INJURY groups exhibiting a significantly higher F/B ratio in the NAÏVE group compared to both SHAM (Kruskal–Wallis *p* = 5.308 × 10^−5^) and INJURY (Kruskal–Wallis *p* = 9.12 × 10^−7^) groups, indicating a shift in dominant phyla composition after cefazolin administration and SCI. *** *p* < 0.001.

The Firmicutes/Bacteroidetes (F/B) ratio is frequently used as a biomarker for gut dysbiosis, with alterations associated with inflammatory states. To further validate microbiome composition shifts, we evaluated the F/B ratio across the experimental groups. Figure 4 shows the NAÏVE group with a significantly higher F/B ratio compared to both SHAM (Kruskal–Wallis *p*= 5.308 × 10^−5^) and INJURY (Kruskal–Wallis *p*= 9.12 × 10^−7^) groups, indicating a marked disruption in the balance of dominant bacterial phyla following cefazolin exposure and/or SCI. Importantly, both SHAM and INJURY animals demonstrated comparable reduced F/B ratios, suggesting that cefazolin alone can significantly perturb the microbiome. These findings support a state of microbial dysbiosis in treated animals, with potential implications for gut barrier function and immune response. * *p* < 0.05, ** *p* < 0.01, *** *p* < 0.001. NAÏVE *n* = 28, SHAM *n* = 15, INJURY *n* = 19.

### 2.5. Temporal Beta Diversity Patterns Reveal Acute Microbial Disruption Post-SCI

To monitor microbial composition shifts across multiple timepoints, beta diversity metrics were evaluated using the Bray–Curtis Dissimilarity Index and visualized through a PCoA.

**Figure 5:**
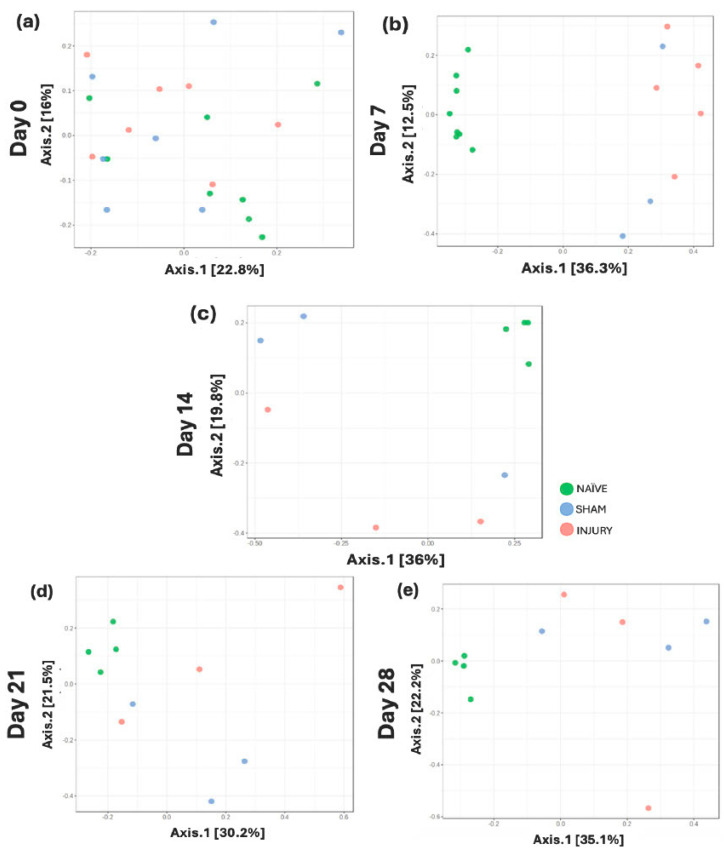
Longitudinal beta diversity measure across five timepoints. PCoA plots based on Bray–Curtis Dissimilarity Index showing microbial community clustering in NAÏVE (green), SHAM (blue), and INJURY (red) groups at days 0, 7, 14, 21, and 28. Minimal group separation was observed at day 0 (**a**) PERMANOVA: NAÏVE vs. SHAM *p*-value = 0.613, NAÏVE vs. INJURY *p*-value = 0.309, SHAM vs. INJURY *p*-value = 0.613, while significant divergence was appreciated at day 7 (**b**) PERMANOVA: NAÏVE vs. SHAM *p*-value = 0.013, NAÏVE vs. INJURY *p*-value = 0.009, SHAM vs. INJURY *p*-value = 0.122. Differences between groups remained statistically significant on day 14 (**c**) PERMANOVA: NAÏVE vs. SHAM *p*-value = 0.049, NAÏVE vs. INJURY *p*-value = 0.049, SHAM vs. INJURY *p*-value = 0.4, day 21 (**d**) PERMANOVA: NAÏVE vs. SHAM *p*-value = 0.044, NAÏVE vs. INJURY *p*-value = 0.044, SHAM vs. INJURY *p*-value = 0.5, and day 28 (**e**) PERMANOVA: NAÏVE vs. SHAM *p*-value = 0.049, NAÏVE vs. INJURY *p*-value = 0.049, SHAM vs. INJURY *p*-value = 0.7, indicating persistent microbial population shifts across time.

At baseline (day 0), no distinct clustering was observed among groups, indicating similar microbial community structures prior to intervention (Figure 5a; PERMANOVA: NAÏVE vs. SHAM *p*-value = 0.613, NAÏVE vs. INJURY *p*-value = 0.309, SHAM vs. INJURY *p*-value = 0.613). By day 7, a clear separation was observed between the NAÏVE group and both SHAM and INJURY groups (Figure 5b; PERMANOVA: NAÏVE vs. SHAM *p*-value = 0.013, NAÏVE vs. INJURY *p*-value = 0.009, SHAM vs. INJURY *p*-value = 0.122), reflecting acute microbial community disruption following cefazolin exposure and SCI. This divergence remained evident on day 14 (Figure 5c; PERMANOVA: NAÏVE vs. SHAM *p*-value = 0.049, NAÏVE vs. INJURY *p*-value = 0.049, SHAM vs. INJURY *p*-value = 0.4), and day 21 (Figure 5d; PERMANOVA: NAÏVE vs. SHAM *p*-value = 0.044, NAÏVE vs. INJURY *p*-value = 0.044, SHAM vs. INJURY *p*-value = 0.5), though with reduced effect. By day 28, significant differences persisted between groups (Figure 5e; PERMANOVA: NAÏVE vs. SHAM *p*-value = 0.049, NAÏVE vs. INJURY *p*-value = 0.049, SHAM vs. INJURY *p*-value = 0.7). This suggests ongoing, though potentially stabilizing, alterations in microbial community structure. Data indicates a sustained but dynamically changing gut microbiota response following cefazolin administration and SCI.

### 2.6. Longitudinal Alpha Diversity Indicates Acute Loss of Richness and Diversity Post-Injury

**Figure 6:**
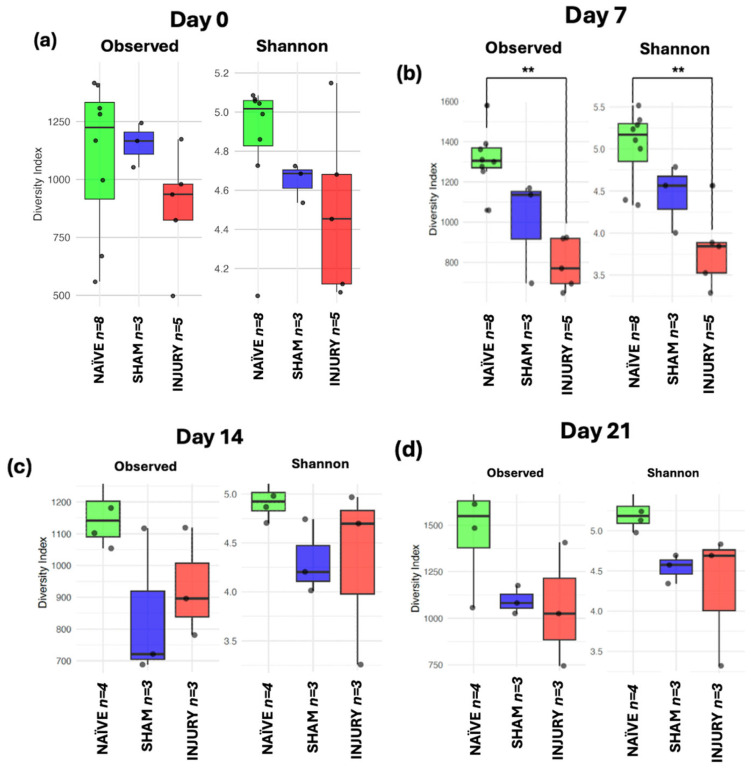

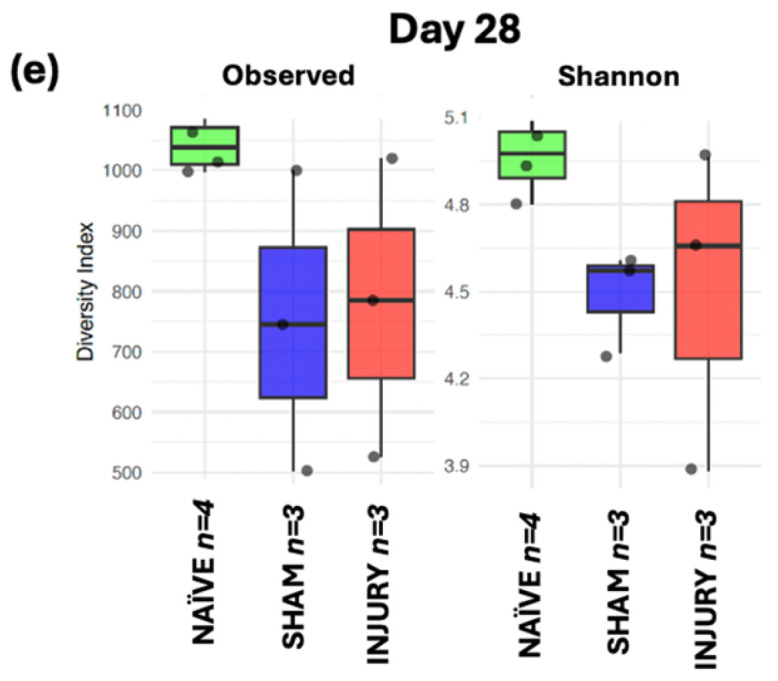
Boxplots showing Observed Richness and Shannon diversity index for NAÏVE, SHAM, and INJURY groups at days 0, 7, 14, 21, and 28. At baseline (day 0) (**a**), no significant differences were detected (Kruskal–Wallis *p* > 0.05, NAÏVE *n* = 8, SHAM *n* = 3, INJURY *n* = 5). On day 7 (**b**), both richness and diversity were significantly reduced in the INJURY group compared to NAÏVE (Kruskal–Wallis *p* < 0.01, NAÏVE *n* = 8, SHAM *n* = 3, INJURY *n* = 5). No significant differences were observed at later timepoints (**c**–**e**) (Kruskal–Wallis *p* > 0.05, NAÏVE *n* = 4, SHAM *n* = 3, INJURY *n* = 3), although NAÏVE rats consistently showed higher alpha diversity metrics. ** *p* < 0.01.

To assess within-sample microbial diversity over time, alpha diversity metrics were used to calculate Observed Richness and Shannon diversity index across five timepoints. At day 0 (Figure 6a), no significant differences were observed among the NAÏVE, SHAM, and INJURY groups in either Observed Richness of Shannon diversity, indicating comparable microbial richness and diversity before any intervention. Importantly, fecal samples collected at day 0 represent baseline microbiota composition prior to cefazolin administration or SCI in all groups. On day 7 (Figure 6b), the INJURY group exhibited a significant reduction in both Observed Richness (Kruskal–Wallis *p* < 0.004) and Shannon diversity (Kruskal–Wallis *p* < 0.008) compared to the NAÏVE, suggesting an acute dysbiotic response following SCI and cefazolin administration. The SHAM group showed a richness and diversity decline but did not differ significantly from the other groups in both metrics (Observed Kruskal–Wallis *p* < 0.124, Shannon Kruskal–Wallis *p* < 0.244) During subsequent timepoints at days 14, 21, and 28 (Figure 6c–e), microbial diversity appeared to partially recover, with no statistically significant differences among the three groups. However, NAÏVE animals still exhibited higher median values in both indices compared to the SHAM and INJURY groups, possibly suggesting a sustained alteration in gut microbiome diversity post-intervention.

### 2.7. F/B Ratio Indicates Transient Phylum-Level Dysbiosis After SCI and Cefazolin Administration

**Figure 7:**
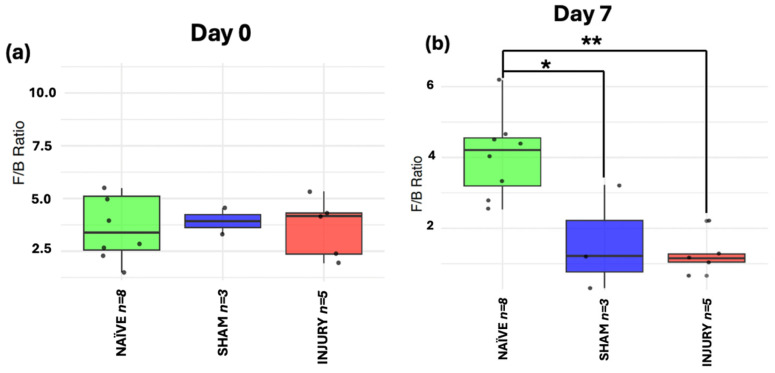

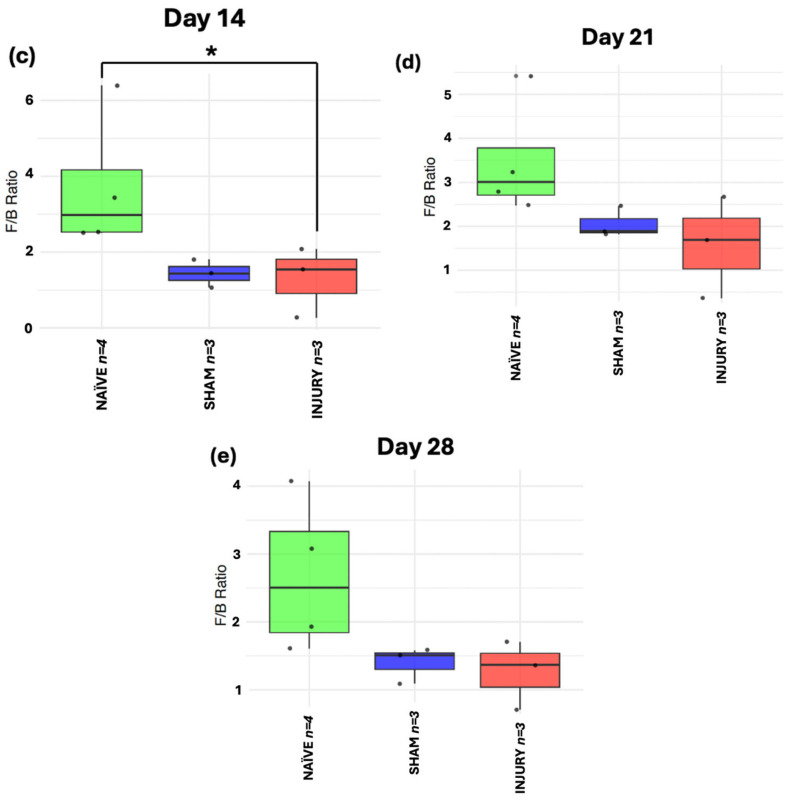
Longitudinal analysis of F/B ratio in NAÏVE, SHAM, and INJURY groups. No significant differences were observed at day 0 (**a**) (Kruskal–Wallis *p* > 0.05, NAÏVE *n* = 8, SHAM *n* = 3, INJURY *n* = 5). The F/B ratio was significantly reduced in the INJURY group compared to NAÏVE at day 7 (**b**) (Kruskal–Wallis *p* < 0.01, NAÏVE *n* = 8, SHAM *n* = 3, INJURY *n* = 5) and day 14 (**c**) (Kruskal–Wallis *p* < 0.05, NAÏVE *n* = 4, SHAM *n* = 3, INJURY *n* = 3), suggesting a transient dysbiosis in the acute phase. No significant differences were observed at later timepoints (**d**,**e**) (Kruskal–Wallis *p* < 0.05, NAÏVE *n* = 4, SHAM *n* = 3, INJURY *n* = 3), although NAÏVE animals maintained the highest F/B ratios throughout the experiment. * *p* < 0.05, ** *p* < 0.01.

The Firmicutes to Bacteroidetes ratio is a metric commonly used as a marker of gut microbiome homeostasis and is often altered in inflammatory conditions. At day 0 (Figure 7a), no significant differences in F/B ratio were observed among groups, indicating stable microbiome composition prior to any intervention (Kruskal–Wallis *p* > 0.05). On day 7 (Figure 7b), both SHAM (Kruskal–Wallis *p* < 0.05) and INJURY group (Kruskal–Wallis *p* < 0.01) exhibited significantly reduced F/B ratios compared to the NAÏVE group, indicating a marked disruption of the microbiota shortly after cefazolin administration and/or SCI. This dysbiotic state was evident until day 14 (Figure 7c) with a significantly lower F/B ratio in the INJURY group (Kruskal–Wallis *p* < 0.05) compared to NAÏVE rats. Microbial differences persisted at days 21 and 28 (Figure 7d,e), although not statistically significant (Kruskal–Wallis *p* > 0.05). The NAÏVE group consistently exhibited higher F/B ratios across the five timepoints, suggesting a more stable microbiome composition. These findings highlight a transient but notable loss of phylum-level balance following the administration of cefazolin and SCI, with the effect further modulated by SCI.

### 2.8. Differential Abundance Taxa Identify Microbial Biomarkers Associated with Injury and Treatment

**Figure 8:**
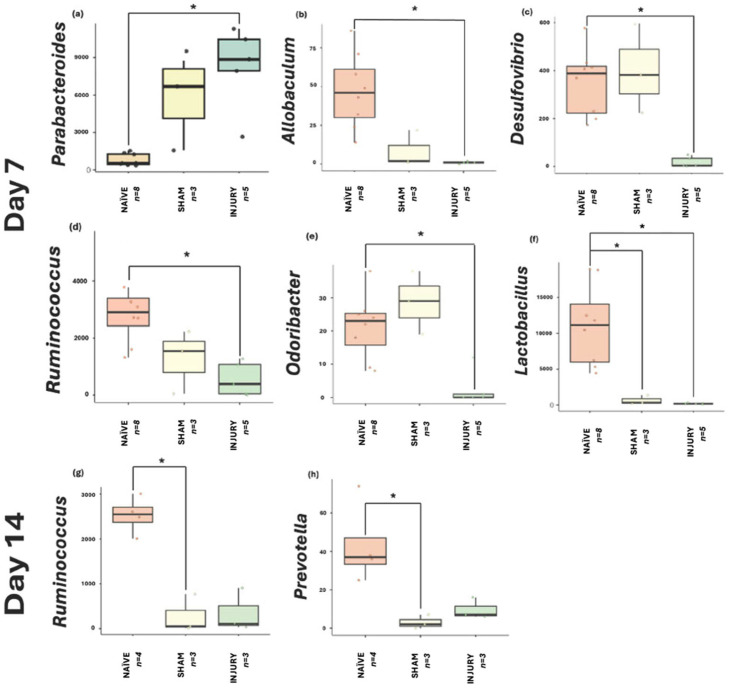
MaAsLin2 boxplots displaying genera significantly associated with INJURY at day 7: (**a**) *Parabacteroides* (FDR = 0.017), (**b**) *Allobaculum* (FDR = 0.020), (**c**) *Desulfovibrio* (FDR = 0.022), (**d**) *Ruminococcus* (FDR = 0.041), (**e**) *Odoribacter* (FDR = 0.042), and (**f**) *Lactobacillus* (FDR = 0.022) (NAÏVE *n* = 8, SHAM *n* = 3, INJURY *n* = 5). The INJURY group displayed significant enrichment of *Parabacteroides* and significant reductions in the remaining genera. Notably, *Lactobacillus* was also substantially depleted in the SHAM group (FDR = 0.040), suggesting cefazolin induces suppression independent of SCI. At day 14, (**g**) *Ruminococcus* (FDR = 0.018) and (**h**) *Prevotella* (FDR = 0.018) were significantly depleted in the SHAM groups compared to NAÏVE controls (NAÏVE *n* = 4, SHAM *n* = 3, INJURY *n* = 3). * *p* < 0.05.

Taxonomic profiles (Supplementary Figures S1–S8) revealed significant compositional shifts in gut microbiome structure across experimental groups and through established timepoints following SCI and cefazolin administration. At the phylum level across experimental groups, NAÏVE rats displayed a higher relative abundance of Firmicutes, reflecting a stable gut microbial environment. In contrast, the INJURY group exhibited enrichment in Proteobacteria and Fusobacteria, along with a decrease in Firmicutes, shifts associated with dysbiosis and inflammation. While these broad community-level changes highlight dysbiosis at the phylum and genus levels, further analysis was conducted to identify specific bacterial taxa associated with treatment effects and SCI. For this purpose, differential abundance using Microbiome Multivariable Associations with Linear Models (MaAsLin2) was performed to identify microbial biomarkers discriminating between experimental groups across the study period. This approach enabled the detection of bacterial biomarkers significantly altered in the INJURY group at day 7, corresponding to the acute post-injury phase (Figure 8a–f). Analysis revealed six genera that were significantly characteristic of the groups. The genus *Parabacteroides* (a) was markedly enriched in the INJURY group relative to both SHAM and NAÏVE (FDR = 0.017), suggesting an early population increase in this genus following the administration of cefazolin and SCI. Several genera commonly associated with gut homeostasis exhibited significantly lower relative abundance in the INJURY group compared to both SHAM and NAÏVE groups, including *Allobaculum* (FDR = 0.020) (b), *Desulfovibrio* (FDR = 0.022) (c), *Ruminococcus* (FDR = 0.041) (d), and *Odoribacter* (FDR = 0.042) (e). Importantly, *Lactobacillus* (f), a genus widely recognized for its probiotic and immunomodulatory effects, showed a significant reduction not only in the INJURY group (FDR = 0.022) but also in the SHAM group (FDR = 0.040), indicating that cefazolin exposure alone may contribute to the depletion of this beneficial genus independent of SCI. Collectively, these findings highlight a dysbiotic microbial signature characterized by the expansion of potentially opportunistic taxa such as *Parabacteroides* and depletion of multiple beneficial commensal bacteria involved in gut barrier integrity, short-chain fatty acid production, and anti-inflammatory regulation.

At day 14 (Figure 8g–h), significant biomarkers were identified primarily associated with cefazolin exposure. *Ruminococcus* (g) and *Prevotella* (h) were significantly reduced in the SHAM group relative to NAÏVE animals (FDR = 0.0187 and FDR = 0.0185, respectively). Although not statistically significant, *Gardnerella* (Supplementary Figure S9) appeared elevated in the INJURY group (FDR = 0.168). At days 21 and 28, statistical analysis did not identify any genera that reached significance. Although compositional shifts were observed in the global taxonomic profiles at earlier timepoints, these differences appeared to diminish over time, with microbial communities showing a trend toward stabilization during the subacute and chronic phases post-injury and cefazolin exposure. Thus, the absence of significant biomarkers at these later timepoints suggests a partial recovery following the initial dysbiosis by SCI and cefazolin administration.

## 3. Discussion

The present study demonstrates that spinal cord injury (SCI) combined with cefazolin administration induces alterations in the gut microbiota of female rats. Using a comprehensive longitudinal design, we observed significant disruptions in microbial diversity and community structure, particularly during the acute post-injury phase, followed by a gradual trend toward partial restoration at later timepoints. These findings contribute to the growing evidence highlighting the systemic consequences of SCI, with the gut microbiome emerging as a potential modulator of post-injury physiology and patient recovery. Moreover, it emphasizes the important effect that antibiotic therapies can have on SCI patients.

In the NAÏVE group, which received no intervention, the gut microbiome remained remarkably stable throughout the entire 28-day observation period. Measures of alpha diversity—including both Observed Richness and Shannon diversity index—were consistently high at all timepoints, and no significant shifts were detected. The Firmicutes/Bacteroidetes (F/B) ratio, a common marker of gut homeostasis, remained high and stable in this group, underscoring the microbiological equilibrium maintained in the absence of any external disturbance. In contrast, the SHAM group, which received cefazolin without spinal cord injury, demonstrated measurable but transient alterations in gut microbiome structure and diversity. While baseline diversity metrics were comparable to those of the NAÏVE group, a notable decline in both richness and diversity was observed by day 7 following cefazolin treatment. Beta diversity showed clear compositional separation between experimental groups in this acute phase, consistent with extensive dysbiosis that occurs following antibiotic exposure and SCI. Consistent with our observations, Schmidt et al. reported a significant decrease in microbial diversity following minocycline administration combined with SCI in rat models, particularly evident five days post-injury, with alterations in both beta and alpha diversity as well as the Firmicutes/Bacteroidetes ratio. Their findings also revealed microbiome stability post-acute phase after SCI and antibiotic administration [37]. These parallels suggest that antibiotic use during the vulnerable early period post-SCI may exacerbate gut microbial disturbances independent of the antibiotic class employed.

At the genus level, our multivariable association analysis through MaAsLin2 identified several taxa significantly altered following SCI and cefazolin administration during the acute post-injury phase. For instance, at day 7, *Parabacteroides* showed a considerable increase in the INJURY group, while key commensal genera such as *Lactobacillus*, *Ruminococcus*, *Allobaculum*, *Desulfovibrio*, and *Odoribacter* were significantly depleted. These bacterial genera are recognized for their roles in gut barrier maintenance, short-chain fatty acid production, and immune homeostasis, suggesting that their depletion may contribute to post-injury dysbiosis and systemic inflammatory responses [38,39,40,41]. At day 14, continued depletion of *Ruminococcus* and *Prevotella* in the SHAM group further signals the sustained dysbiotic effect of cefazolin even in the absence of SCI. No significant biomarkers were detected on days 21 or 28, consistent with partial microbial recovery observed at these later stages. Lastly, the decrease in *Lactobacillus* observed in our study is consistent with clinical findings reported by Li et al., who described microbiome alterations at the genus level in patients with acute SCI receiving prophylactic and therapeutic antibiotics, including the enrichment of *Eryspelotrichaceae*, *Lachnospiraceae*, and *Lachnoclostridium* [42]. Together, these genus-level disruptions underscore the converging microbial signatures that emerge in both research and clinical settings after SCI and antibiotic treatment, emphasizing the need to balance infection management with the preservation of microbiome stability during SCI care.

Beyond taxonomic shifts, several studies have proposed that gut microbiome alterations following SCI may contribute to the development of systemic inflammation, metabolic dysfunction, and impaired immune interactions [43]. Gut dysbiosis may disrupt intestinal epithelial integrity, increase gut permeability, and facilitate translocation of microbial products such as lipopolysaccharide (LPS) into systemic circulation, thereby triggering chronic inflammation. Inflammatory cytokine production may further impact neural repair mechanisms, exacerbate neuropathic pain, and influence metabolomic dysregulation commonly observed after SCI [44,45]. In this context, maintaining microbial stability during the acute phase following SCI is proposed to have therapeutic relevance beyond infection alone.

Taken together, these findings reinforce the concept that SCI triggers a cascade of systemic disturbances extending beyond the central nervous system. The gut microbiome may serve as both a mediator and consequence of this dysregulation, with antibiotic use further aggravating microbial instability. While prophylactic antibiotics remain a standard and warranted clinical practice for infection prevention during the acute phase of SCI, our results—alongside prior research and clinical studies—suggest that careful consideration of antibiotic regimens is justified to minimize long-term dysbiosis consequences. It is important to note that our study is limited by the relatively small sample size in the SHAM group (*n* = 3), as well as a reduced number in the INJURY group at later timepoint (*n* = 3 on days 14, 21 and 28), which may constrain statistical power and affect the results, and the absence of a SCI only group untreated with antibiotics, which restricts the ability of isolate the effects of SCI from those of cefazolin treatment. Despite these limitations, the trends observed provide meaningful insights and warrant further investigation. The partial continuation of shifts at Day 14 (especially in the SHAM group) supports the conclusion that cefazolin alone can cause sustained gut microbial alterations, which could interfere with immune modulation and recovery if not managed therapeutically. Future studies with larger cohorts and extended sampling periods are necessary to clarify these interactions. To evaluate whether targeted microbiome-based interventions—such as pre- and probiotic supplementation—in male and female rats could mitigate gut dysbiosis and promote improved recovery outcomes after SCI.

## 4. Materials and Methods

### 4.1. Animal Housing

Adult female Sprague Dawley rats (∼230 g) were purchased from Hilltop Lab Animals (Scottdale, PA, USA). Animals were pair-housed during acclimation and individually housed throughout the experimental phase and post-operative care [46]. Environmental conditions were maintained under a 12:12 h light–dark cycle, with food chow (Harlan Teklad) and water provided ad libitum. All procedures, including surgeries and post-operative care, were reviewed and approved by the Institutional Animal Care and Use Committee (IACUC) of the University of Puerto Rico, Medical Sciences Campus.

### 4.2. Laminectomy, Spinal Cord Injury (SCI), and Drug Administration

Surgical procedures were conducted under sterile and aseptic conditions. A total of 16 rats were included in the study, with 8 animals undergoing surgery, and 8 served as NAÏVE controls, receiving no surgical intervention or drug treatment. Moderate compression of the spinal cord was confirmed with a Basso, Beattie, and Bresnahan (BBB) score ≤ 2, assessed two days post-injury. All surgical procedures were performed following previously published protocols [47,48]. Briefly, anesthesia was induced with a ketamine/xylazine/acepromazine cocktail 36/3.6/1.6 mg/mL. Animals were randomly assigned to either the NAÏVE, INJURY, or SHAM group. SHAM animals underwent a laminectomy at the T-10 vertebral level, followed by immediate suturing of the incision. In the SCI group, a laminectomy at T-10 was followed by a moderate contusion using the NYU/MACSIS impactor device. The vertebral column was stabilized with clamps, and a 10 g rod was dropped from a height of 12.5 mm onto the exposed spinal cord, with compression sustained for 5 s. Post-operative care included suturing the surgical site and subcutaneous administration of saline. To prevent corneal dryness, eyedrops were applied, and cefazolin (25 mg/kg) was administered intraperitoneally twice daily for 7 days as antibiotic prophylaxis. This dosing schedule was based on previous experiments conducted in our laboratory, where a 7-day regimen proved effective in minimizing postoperative infections in the acute post-injury phase. Analgesia was provided with buprenorphine (0.05 mg/kg) twice daily for 3 days. Manual bladder expression was performed for the injury group until micturition reflexes resumed [49]. Cereal and paper-based enrichment materials were provided throughout the experiment.

### 4.3. Rat Fecal Matter Collection

Approximately 250 mg of fecal matter was collected aseptically using sterilized forceps washed in alcohol between each animal to prevent cross-contamination. Samples were collected one week before experimental procedures (day 0), one week after surgeries (day 7), followed by seven-day intervals until day 28 (Figure 1).

### 4.4. Genomic DNA Extraction

Genomic DNA (gDNA) was extracted from rat fecal matter pellets with the Dneasy PowerSoil Kit (QIAGEN, Germantown, MD, USA) following the manufacturer’s protocol. Briefly, 800 μL of CD1 solution was added to the PowerBead Pro Tubes© (QIAGEN, Germantown, MD, USA) along with the 250 mg of fecal matter. Sample homogenization was achieved by placing the PowerBead Pro Tubes© horizontally in the vortex and mixing at maximum speed for 10 min. Two hundred microliters of CD2 solution were added to precipitate non-DNA organic and inorganic material, like humic substances, cell debris, and proteins. Then, 600 μL of CD3 solution was added to each tube. CD3 contains high salt concentrations, helping to bind the DNA tightly to silica beads. Then, 500 μL of the EA wash buffer was added to each sample to remove protein and other non-aqueous contaminants; further wash was achieved by adding 500 μL of ethanol-based CD5 solution. Lastly, elution was made with 50 μL of CD6 solution, and approximately 30 μL of DNA was eluted from each sample. gDNA quantifications were performed using the Qubit 1 X dsDNA HS Assay Kit (high sensitivity; Thermo Fisher, Waltham, MA, USA) and the Qubit Flex Fluorometer. The 16S rRNA gene hypervariable V4 region was amplified using the universal primers 515F (5′-TGCCAGCMGCCGCGGTAA-3′) and 806R (5′- GGACTACHVGGGTWTCTAAT- 3′). Amplicons were sequenced on Illumina MiSeq (Illumina, San Diego, CA, USA)using a 2 × 250 base pairs (bp) paired-end sequencing protocol, a run configuration where each DNA fragment is sequenced from both ends, producing two reads of 250 bp each. Quality control for 16S rRNA demultiplexes amplicons was achieved in QIITA (v2025.07) [50] and analyzed with the platforms QIIME2 (v.2023.7) [51], R (v4.3.1) [48], and MicrobiomeAnalyst (v4.0) [49].

### 4.5. Microbial Community Analyses

#### 4.5.1. Beta Diversity

Ordination plots were calculated using the web-based platform MicrobiomeAnalyst [49]. A variant-based diagram was constructed using resilient Principal Coordinates Analysis (PCoA), allowing the association of specific features or taxa with the beta diversity ordination. Each point on the plot represents an individual operational taxonomic unit (OTU). Permutational multivariate analysis of variance (PERMANOVA) based on the Bray–Curtis Dissimilarity Index was performed to assess the statistical significance of group differences in microbial composition.

#### 4.5.2. Alpha Diversity, Taxonomic Profiles, and Microbial Biomarkers

Alpha diversity metrics were assessed using the Observed Richness and Shannon diversity indices to evaluate microbial richness and diversity, respectively. Visualizations were generated using the phyloseq [50], vegan [51], and ggplot2 [52] packages in R [48]. Statistical significance was determined using the non-parametric Kruskal–Wallis test followed by Dunn’s post hoc. Furthermore, bar plots depicting taxonomic composition at the phylum and genus levels were generated to illustrate relative abundance patterns across groups using MicrobiomeAnalyst [48] (Supplementary Figures S2 and S4).

To identify bacterial genera associated with the pro-inflammatory effects of cefazolin and SCI, differential abundance analysis was conducted using MaAsLin2 [52] library in R. Genera with a q-value < 0.05 were considered significant and visualized using boxplots. Additionally, the LEfSe (Linear Discriminant Analysis Effect Size) algorithm was applied in MicrobiomeAnalyst to detect discriminatory taxa among the three experimental groups. Taxa with an LDA score ≥ 1.0 and a *p*-value < 0.05 were considered significant. Heatmaps were generated to visualize taxa abundance across all five timepoints (Supplementary Figures S1–S4).

#### 4.5.3. F/B Ratio

As mentioned, the Firmicutes/Bacteroidetes (F/B) ratio is increasingly recognized as a potential biomarker for dysbiosis in microbiome studies. To evaluate this metric, boxplots of the F/B ratio were generated using the ggpubr package (53) in R, along with tidyverse for data analysis, and statistical significance was assessed using the non-parametric Kruskal–Wallis test followed by Dunn’s post hoc.

## 5. Conclusions

Before presenting our concluding remarks, it is essential to acknowledge the limitations inherent in this study. A primary limitation was the absence of a spinal cord injury (SCI) group that did not receive cefazolin, a decision dictated by the ethical and welfare constraints of our IACUC-approved protocol. This restriction was implemented to minimize animal distress and prevent mortality associated with untreated postoperative infections. Nevertheless, our extensive laboratory experience consistently demonstrates the critical role of antibiotic administration in mitigating animal loss following SCI. Moreover, the design of the experimental groups was intended to closely mirror real-world clinical scenarios, in which human patients presenting to emergency departments routinely receive antibiotic prophylaxis as part of standard care protocols. In addition, the number of animals included in each experimental group was limited by the availability of subjects meeting the predefined inclusion criteria at the time of study initiation. Although smaller cohort sizes inevitably constrain statistical power, this limitation was mitigated by the application of deep 16S rRNA sequencing, which enabled high-resolution taxonomic profiling and enhanced the robustness and interpretive value of the microbial community data. Similar constraints are well-documented in microbiota research, particularly in studies involving protected species or ethically sensitive models, where legal, welfare, and regulatory considerations necessarily take precedence over experimental scalability. Consequently, while the findings of this study should be interpreted within the context of these ethical and methodological boundaries, the data generated remain biologically meaningful. Furthermore, the insights derived from this work provide a valuable foundation for future investigations employing larger cohorts, which will be critical for validating and expanding upon the conclusions presented here.

In conclusion, in this study, we demonstrate that cefazolin antibiotic induces significant alterations in the gut microbiome composition in female rats that were potentiated after SCI, especially during the acute post-injury phase. Decrease in microbial diversity, enrichment of potentially pro-inflammatory taxa, and depletion of key commensal genera were observed shortly after the injury, with partial microbial population diversity occurring at later stages after the injury. Although the microbiome of the SHAM animals showed trends toward partial restoration in diversity at later stages, no significant differences were detected, and the findings should be interpreted with caution due to the limited sample size. While the use of antibiotics remains clinically warranted, principally in the acute phase of SCI to prevent and manage life-threatening infections, our findings emphasize the collateral impact of such treatments may have on gut microbial homeostasis. These results highlight the critical interplay between SCI, antibiotic administration, and gut microbiome dynamics. This suggests that strategies aimed at preserving microbiome stability may represent a valuable approach to optimize recovery and minimize systemic complications after SCI. Moreover, neuroprotective and/or neuroregenerative therapies should be drugs that reduce dysbiosis induced by SCI and antibiotic treatment, or medications that promote gut diversity. Future studies using larger sample sizes will be necessary to validate these findings and better define any sex specific microbiome-based interventions.

## Data Availability

The 16S rRNA gene sequences can be found in the QIITA study ID 16026. They are also available in the European Nucleotide Archive ENA Project (PRJEB90933, ERP173928).

## Supplementary Materials

The following supporting information can be downloaded at: https://www.mdpi.com/article/10.3390/microorganisms13102324/s1, Figure S1: Heatmap of microbial composition at the phylum level; Figure S2: Heatmap of microbial composition at the genus level; Figure S3: Heatmap of phylum-level microbial composition across five time points (Days 0, 7, 14, 21, and 28); Figure S4: Heatmap of genus-level microbial composition across time points; Figure S5: Stacked bar plot of phylum-level relative abundance across experimental groups; Figure S6: Stacked bar plot of genus-level relative abundance across experimental groups; Figure S7: Stacked bar plot of phylum-level relative abundance across time points; Figure S8: Stacked bar plot of genus-level relative abundance across time points; Figure S9: Relative abundance of the genus Gardnerella across experimental groups at day 14 post-injury.
