# Supplementary material for: The Effect of Cefazolin on the Gut Microbiome of Female Rats After Spinal Cord Injury
Source: Microorganisms. 2025 Oct 7;13(10):2324. doi: 10.3390/microorganisms13102324 (PMC12566097; doi:10.3390/microorganisms13102324)
Supplement: Supplementary file 1 [file microorganisms-13-02324-s001.zip › microorganisms-3892071-supplementary.pdf]

Supplementary Figure S1

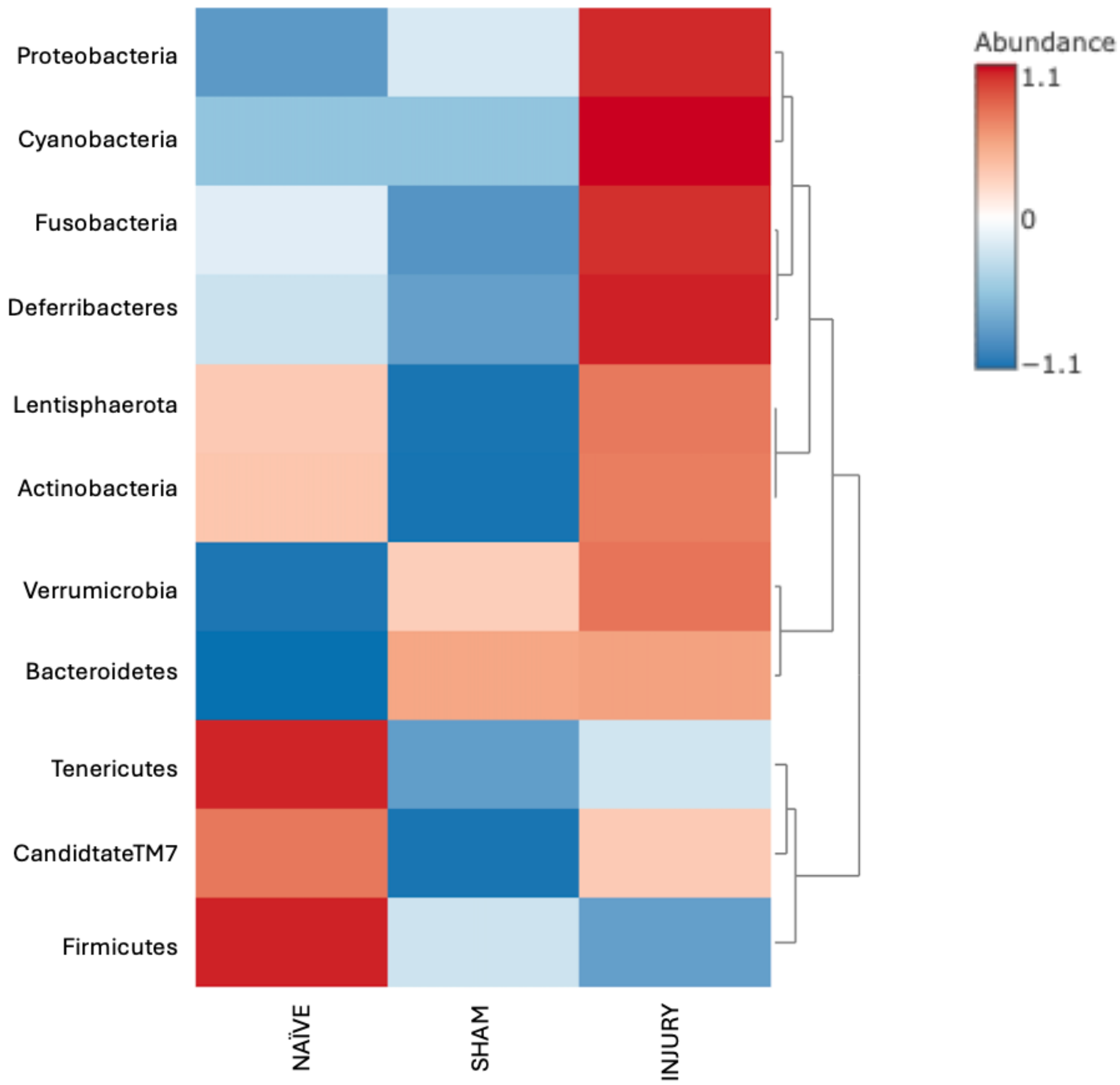

Supplementary Figure S1. Heatmap of microbial composition at the phylum level.
Heatmap displaying relative abundances of dominant phyla in NAÏVE, SHAM, and INJURY groups. Clustering was performed using Euclidean distance and Ward's linkage algorithm in MicrobiomeAnalyst. Distinct shifts in phylum composition are observed across groups, with notable enrichment of Proteobacteria and Cyanobacteria in the INJURY group.

Supplementary Figure S2

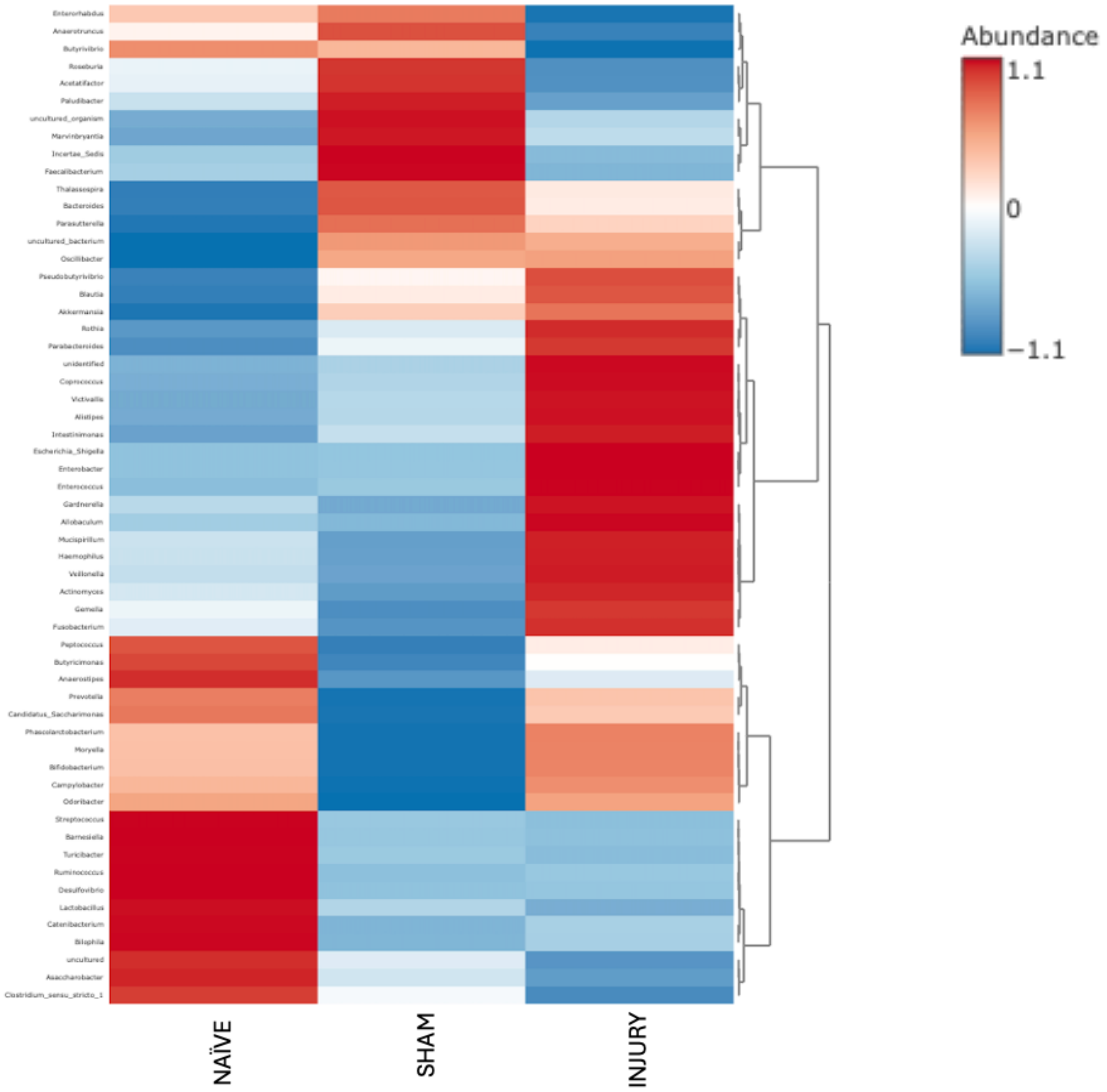

Supplementary Figure S2. Heatmap of microbial composition at the genus level.
Heatmap illustrating relative abundances of microbial genera across NAÏVE, SHAM, and INJURY groups. Genera display distinct clustering patterns according to treatment group, highlighting substantial shifts in gut microbial composition following spinal cord injury and cefazolin administration. Heatmap was generated using Euclidean distance and Ward's linkage algorithm.

Supplementary Figure S3

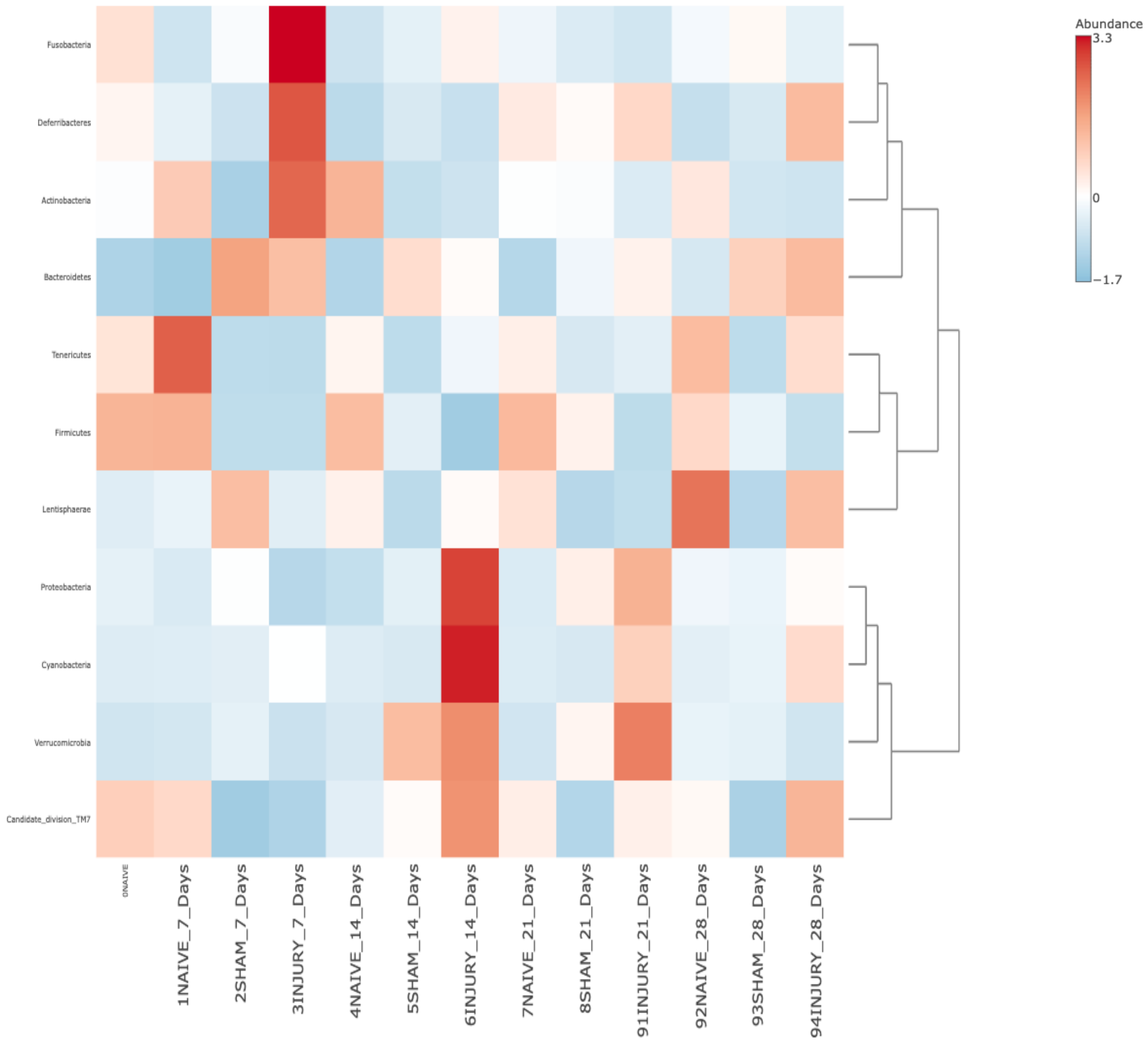

Supplementary Figure S3. Heatmap of phylum-level microbial composition across five time points (Days 0, 7, 14, 21, and 28). INJURY animals exhibited marked phylum shifts during the acute phase, most pronounced at Days 7 and 14.

Supplementary Figure S4

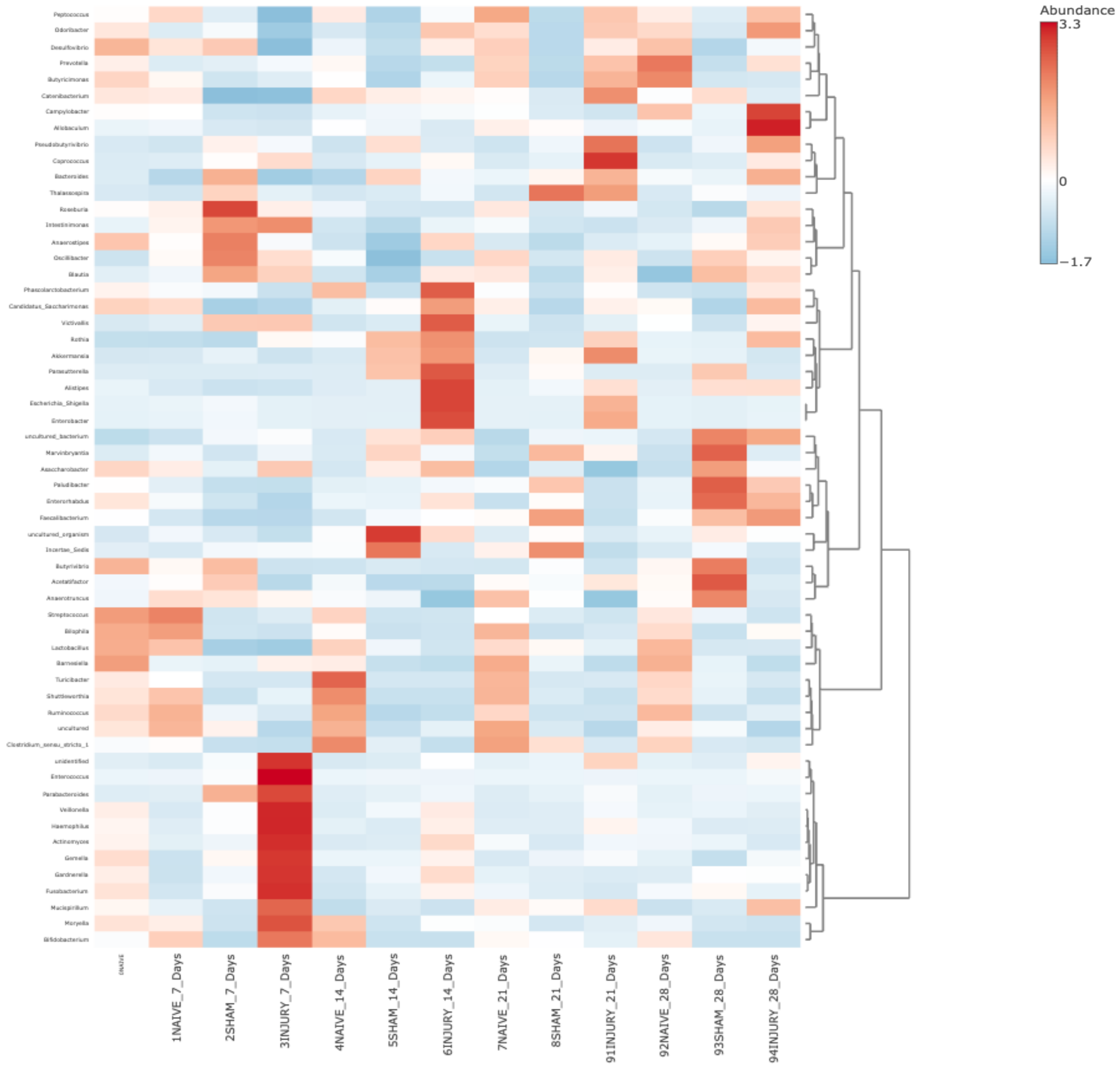

Supplementary Figure S4. Heatmap of genus-level microbial composition across time points. Temporal dynamics highlight acute shifts in INJURY and SHAM groups compared to NAÏVE.

Supplementary Figure S5

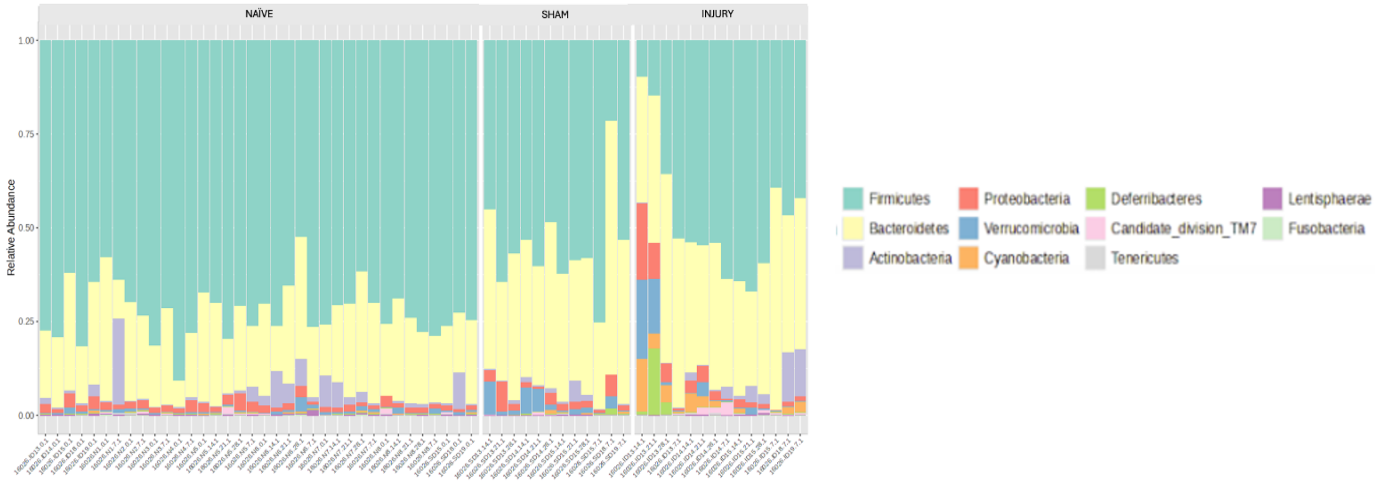

Supplementary Figure S5. Stacked bar plot of phylum-level relative abundance across experimental groups..

Supplementary Figure S6

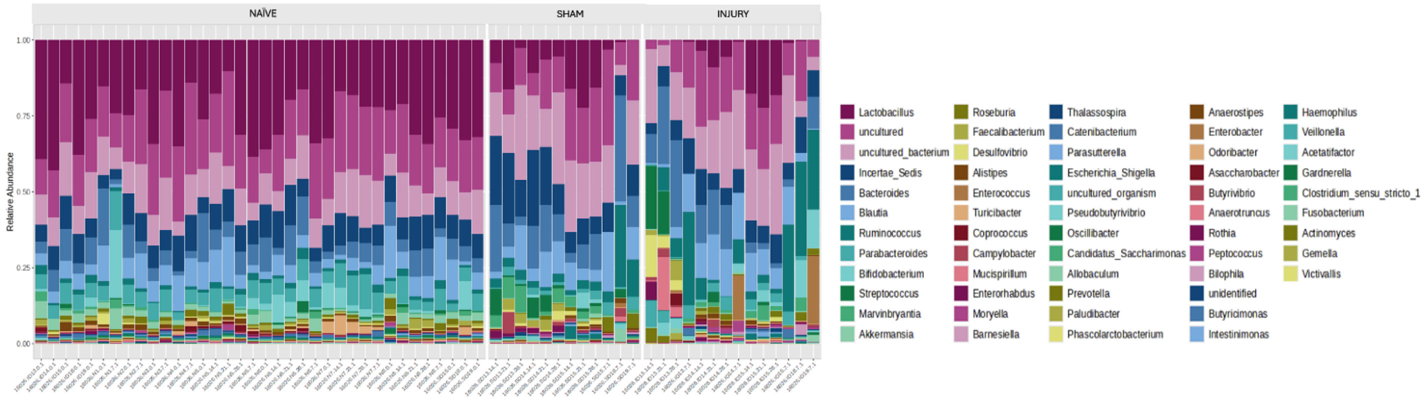

Supplementary Figure S6. Stacked bar plot of genus-level relative abundance across experimental groups.

Supplementary Figure S7

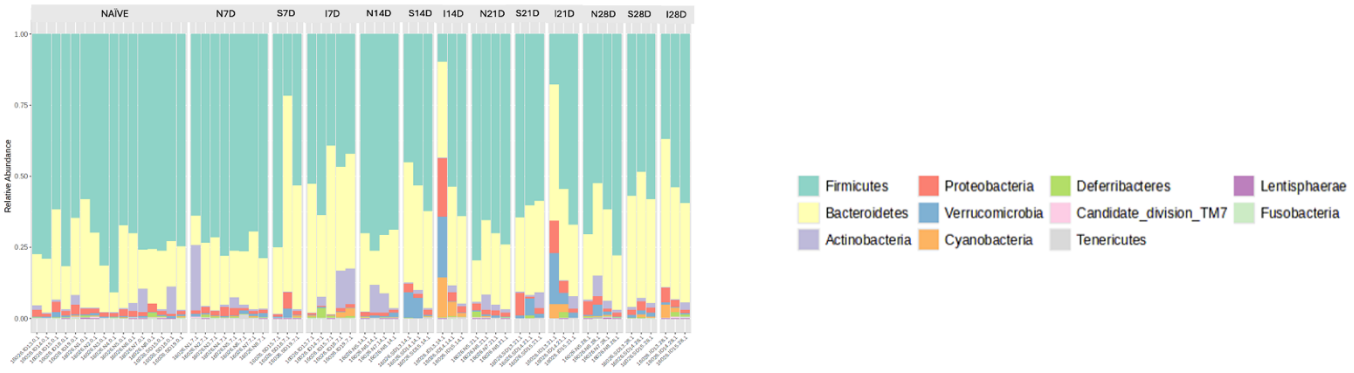

Supplementary Figure S7. Stacked bar plot of phylum-level relative abundance across time points.

Supplementary Figure S8

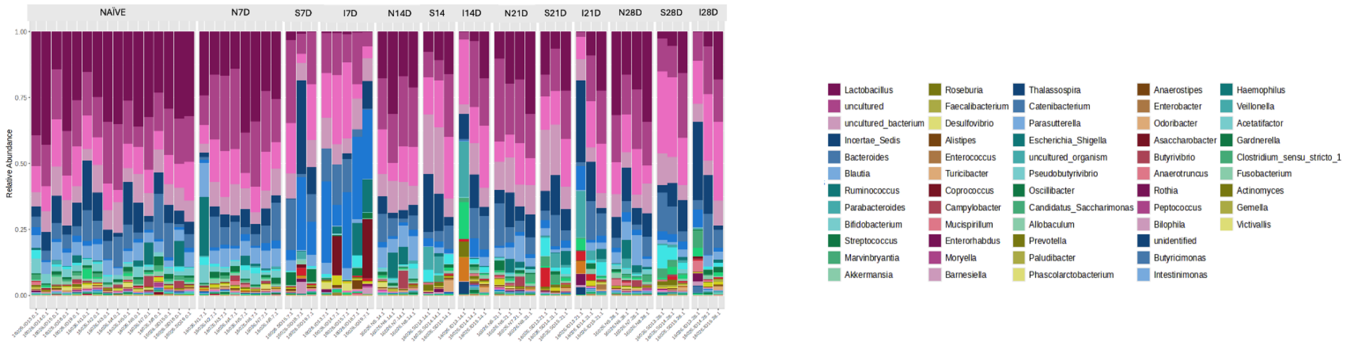

Supplementary Figure S8. Stacked bar plot of genus-level relative abundance across time points.

Supplementary Figure S9

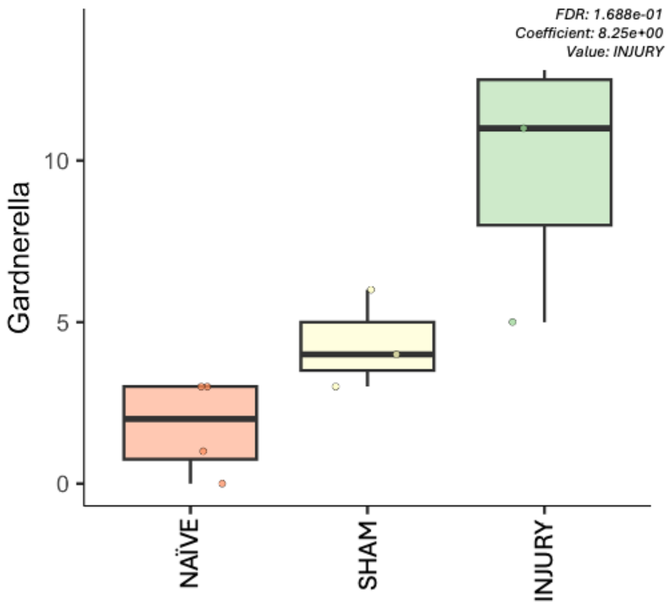

Supplementary FigureS9. Relative abundance of the genus Gardnerella across experimental groups at day 14 post-injury. *Boxplots illustrate increased abundance of Gardnerella in the INJURY group compared to the NAÏVE and SHAM groups. Statistical analysis using MaAsLin2 identified this increase as non-significant (FDR = 0.169, coefficient = 8.25). Despite not reaching statistical significance, a progressive elevation in Gardnerella abundance is observed from NAÏVE to SHAM and INJURY groups, suggesting a possible association with spinal cord injury and cefazolin administration.*
